# A radiomics approach to the diagnosis of femoroacetabular impingement

**DOI:** 10.3389/fradi.2023.1151258

**Published:** 2023-03-20

**Authors:** Eros Montin, Richard Kijowski, Thomas Youm, Riccardo Lattanzi

**Affiliations:** ^1^ Bernard and Irene Schwartz Center for Biomedical Imaging, Department of Radiology, New York University Grossman School of Medicine, New York, NY, United States; ^2^ Center for Advanced Imaging Innovation and Research (CAI^2^R), Department of Radiology, New York University Grossman School of Medicine, New York, NY, United States; ^3^ Department of Radiology, New York University Grossman School of Medicine, New York, NY, United States; ^4^ Department of Orthopedic Surgery, New York University Grossman School of Medicine, New York, NY, United States

**Keywords:** radiomic, MRI, machine learning and AI, femoroacetabular impingement syndrome, features & signature, kNN (k nearest neighbor), hip joint, automatic diagnosis and prediction models

## Abstract

**Introduction:**

Femoroacetabular Impingement (FAI) is a hip pathology characterized by impingement of the femoral head-neck junction against the acetabular rim, due to abnormalities in bone morphology. FAI is normally diagnosed by manual evaluation of morphologic features on magnetic resonance imaging (MRI). In this study, we assess, for the first time, the feasibility of using radiomics to detect FAI by automatically extracting quantitative features from images.

**Material and methods:**

17 patients diagnosed with monolateral FAI underwent pre-surgical MR imaging, including a 3D Dixon sequence of the pelvis. An expert radiologist drew regions of interest on the water-only Dixon images outlining femur and acetabulum in both impingement (IJ) and healthy joints (HJ). 182 radiomic features were extracted for each hip. The dataset numerosity was increased by 60 times with an ad-hoc data augmentation tool. Features were subdivided by type and region in 24 subsets. For each, a univariate ANOVA *F*-value analysis was applied to find the 5 features most correlated with IJ based on *p*-value, for a total of 48 subsets. For each subset, a K-nearest neighbor model was trained to differentiate between IJ and HJ using the values of the radiomic features in the subset as input. The training was repeated 100 times, randomly subdividing the data with 75%/25% training/testing.

**Results:**

The texture-based gray level features yielded the highest prediction max accuracy (0.972) with the smallest subset of features. This suggests that the gray image values are more homogeneously distributed in the HJ in comparison to IJ, which could be due to stress-related inflammation resulting from impingement.

**Conclusions:**

We showed that radiomics can automatically distinguish IJ from HJ using water-only Dixon MRI. To our knowledge, this is the first application of radiomics for FAI diagnosis. We reported an accuracy greater than 97%, which is higher than the 90% accuracy for detecting FAI reported for standard diagnostic tests (90%). Our proposed radiomic analysis could be combined with methods for automated joint segmentation to rapidly identify patients with FAI, avoiding time-consuming radiological measurements of bone morphology.

## 1. Introduction

Femoroacetabular impingement (FAI) is a common cause of hip pain in young adults with an estimated incidence of 54.4 per 1,00,000 person-years (1). FAI is characterized by impingement of the femoral head-neck junction against the acetabular rim during hip joint motion due to morphologic abnormalities of the proximal femur and acetabulum (2–4). There are two distinct pathoanatomic types of FAI, although mixed types are commonly detected at arthroscopy (5). Cam FAI is caused by decreased offset and asphericity of the femoral head-neck junction, while Pincer FAI is due to focal or generalized acetabular over-coverage (3, 4). Although the natural history of FAI is unknown, early diagnosis and appropriate surgical treatment of the condition has been shown to reduce symptoms and improve function, at least in the short-term (6).

Imaging plays an important role in the diagnosis of FAI as distinguishing the disorder from other causes of hip pain is challenging using clinical history and physical examination (7). Quantitative measures of bone shape on radiographs including the alpha angle for Cam impingement and the center edge angle for Pincer impingement are typically used for the initial diagnosis of FAI (3, 4). However, radiographic measures of bone shape may be influenced by technical factors during image acquisition (8–10), and three-dimensional (3D) bone morphology may not be reliably assessed on two-dimensional (2D) radiographs (11, 12). Thus, computed tomography (CT) is commonly used for pre-operative planning to provide the most accurate assessment of 3D bone shape (3, 4). While CT provides high spatial resolution and excellent tissue contrast for evaluating bone, it may result in potentially harmful ionizing radiation exposure to the pelvis (13).

Recent literature (3, 4, 14, 15) focused the attention of FAI diagnosis on 3D MR imaging, which can enable radiologists to detect the typical osseous pathological condition in FAI with accuracy, sensitivity and specificity around 90% (3, 4). These analyses are usually based on metrics arising from the shape of the hip structures or from range of motion simulations of the hip joint (6, 7, 15–17).

Radiomics has gained increasing popularity over the recent years as a diagnostic image analysis method to predict and characterize a wide variety of pathologic conditions (18–22). Radiomics involves the high-throughput extraction of quantitative features from medical imaging studies such as CT and MRI (19–21). The assumption of radiomics is that image features quantify crucial information regarding pathologic conditions through intra-region heterogeneity (19). Several studies have used radiomics to evaluate musculoskeletal diseases of soft tissue and bone (23). However, to our knowledge no previous work has investigated the use of radiomics to diagnose FAI (24). Thus, our study was performed to investigate the feasibility of using radiomics on 3D-MRI to distinguish between hips with and without symptomatic impingement in patients with FAI.

## 2. Material and methods

### 2.1. Image data

The study group consisted of 17 patients (13 females and four males with mean age of 37.1 ± 5.7 years) with unilateral FAI diagnosed at hip arthroscopy who underwent an MRI examination of the hip prior to surgery. One patient was diagnosed with isolated Cam FAI, while the remaining 16 patients were diagnosed with mixed Cam and Pincer FAI at arthroscopy. Three patients underwent a follow-up MRI examination one year after surgery. All MRI examinations were performed on a 3T scanner (Skyra, Siemens Healthineers, Erlangen, Germany) and included an axial dual echo T1-weighted 3D fast low angle shot (FLASH) sequence of the pelvis with Dixon fat-water separation and the following imaging parameters: repetition time = 10 ms, echo time = 2.4 ms and 3.7 ms, field of view = 32 cm, acquisition matrix = 320 × 320, and slice thickness = 1 mm.

For each MRI dataset, a fellowship-trained musculoskeletal radiologist with 20 years of clinical experience delineated regions of interest (ROIs) for the femur and acetabulum on each water-only image slice of the 3D-FLASH sequence using an open-source software viewer (ITK-SNAP v3.8.0; www.itksnap.org) (25).The ROIs were drawn using the automatic 3D seed based segmentation tool available in ITK-SNAP and then manually fine-tuned slice by slice in the three main visualization axes: axial, coronal, and sagittal.

**Figure 1:**
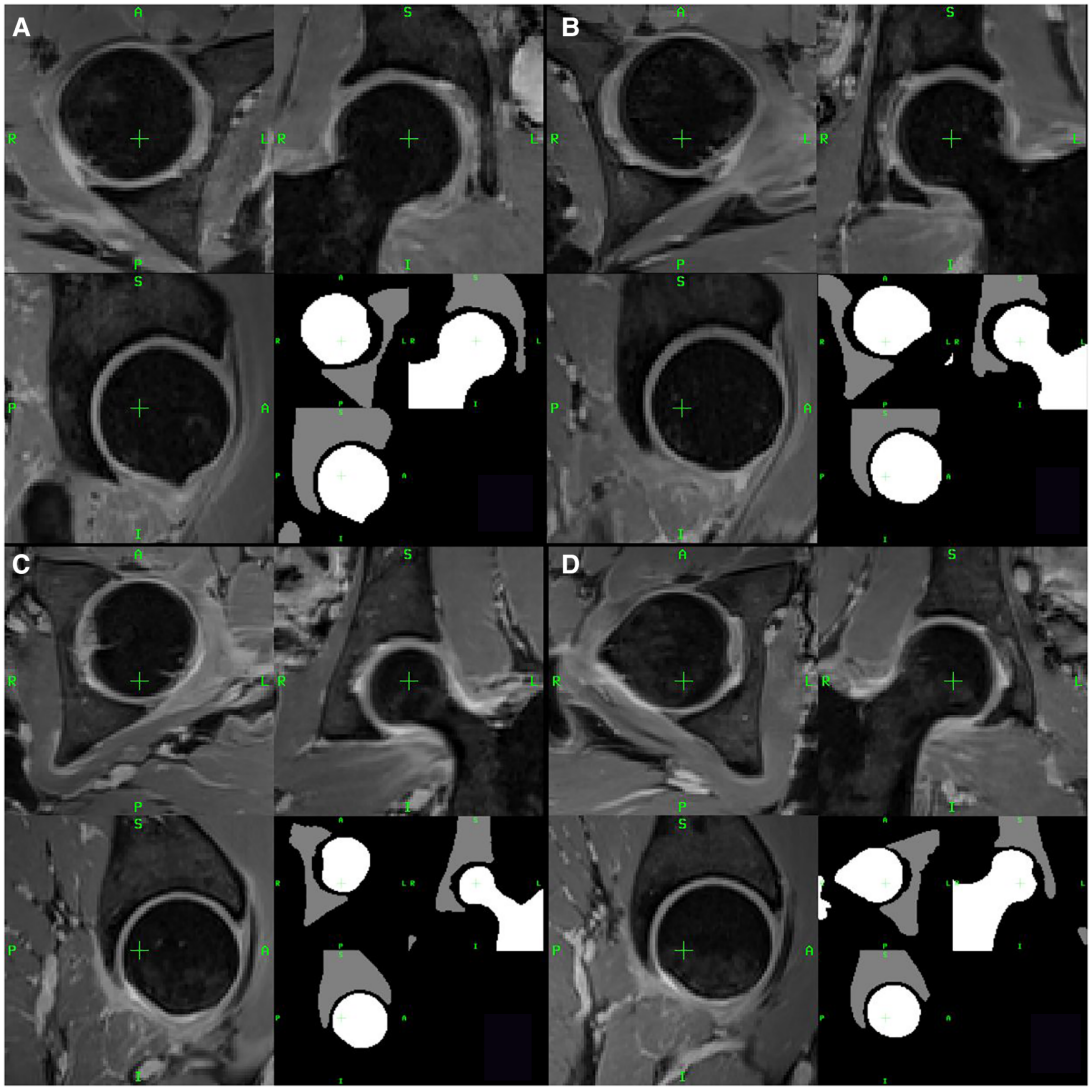
Two examples of healthy joints (**A**,**B**) and two examples of joints with impingement (**C**,**D**) from two representative patients with FAI. For each example, axial, coronal and sagittal views are shown. The lower right quadrant of each panel shows the segmented femur (white) and acetabulum (gray) ROIs.

Left and right hip femur and acetabulum ROIs for the 17 patients were subdivided into healthy joints (HJs) and joints with impingement (IJs) according to the surgical reports. The IJs of the three patients with follow-up MRI examinations were excluded as the femur and acetabulum were surgically remodeled during arthroscopy. This resulted in a total of 37 segmented femoral and acetabular ROIs, which included 17 HJs and 17 IJs from the pre-operative MRI examinations and three HJs from the post-operative MRI examinations. Figure 1 shows representative examples of segmented femoral and acetabular ROIs from HJs and IJs.

### 2.2. Data augmentation

To increase sample size, a data augmentation method was used that provided rototranslated couples of images and ROIs that were sampled at different resolutions. Directly applying rototranslation and subsequently changing the resolution of the image could result in erroneously labeled pixels in the transformed ROIs due to the interpolation process after the rototranslation, or pixels affected by partial volume averaging. The developed data augmentation technique instead transformed every label map ROI in a collection of meshes, one per value of the map, and then transformed them along with the corresponding image. The transformations were applied in the non-gridded space of the meshes and then rasterized in the desired space. The output coordinate system could be also customized by setting origin, direction, resolution, and size of the output grid space. Data augmentation was implemented in ITK4 (26) and a containerized version of the software has been made freely available at https://hub.docker.com/r/erosmontin/daug.

**Figure 2:**
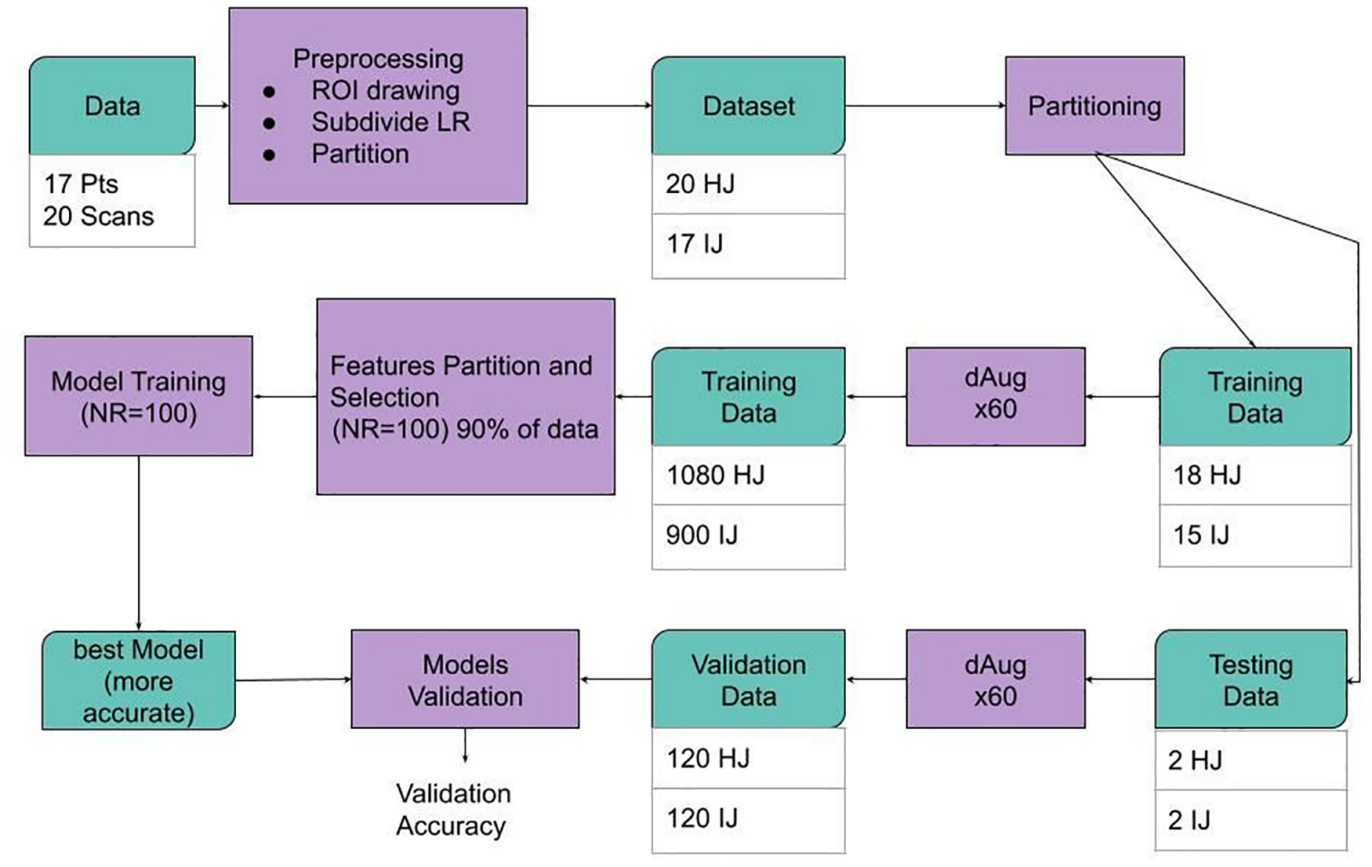
Schematic representation of the data workflow. Data was pre-processed, and images and regions of interest (ROIs) from a total of 3 datasets [18 healthy joints (HJs) and 15 joints with impingement (IJs)] were used for the model training phase, while four hold-out testing datasets (two healthy joints and two joints with impingement) were used for model evaluation. The size of the training and validation datasets was augmented by a factor of 60 using a data augmentation (dAug) method. 48 subsets of features were created from randomly selected 75% of the training data. For each subset of features, a KNN machine learning process was repeated 100 times and the most accurate model was selected for each case. Finally, the performance of the best model for each subset was assessed on the hold-out testing dataset.

As described in the workflow diagram in Figure 2, the 37 labeled and segmented femur and acetabulum ROIs were augmented by a factor of 60 for a total of 2,220 datasets. The 2,220 augmented datasets were obtained by creating randomly uniformed rototranslation between −5 and 5° in the first two Euler’s angles (left/right and anterior/posterior) and between −15 and 15° in the third Euler’s angle (inferior/superior), with random translations ranging between 5 and −5 mm. The resulting images were re-sampled using two output coordinate systems: a uniform grid of 1 mm side and a size of 120 voxels per dimension and an anisotropic grid of resolution 0.4 × 0.4 × 1.2 mm and matrix size of 320 × 320 × 120. In order to maintain the anatomical shape of the hips as realistic as possible, no scaling was applied to the datasets.

### 2.3. Radiomic features extraction

For each couple consisting of an image and one associated femur or acetabulum ROI in the augmented dataset, 182 features were extracted using a previously described radiomic feature extractor (19), including 91 features for the femur and 91 features for the acetabulum. The 91 features could be classified into three main classes: (i) intensity and histogram based first order statistics (FOS) features, (ii) texture features, and (iii) shape and size features. A complete list of the 91 features extracted from the augmented datasets is summarized in Table 1.

**Table 1:** List of radiomic features. For each feature, the *p*-value of the Wilcoxon rank-sum test is reported along with the mean values for the feature distribution for the health joints (HJs) and joints with impingement (IJs). Gray cells are associated with a statistically significant difference between the distribution of the feature values in the HJs and IJs. SS, shape and size; 3D, three-dimensional; GLCM, gray level co-occurrence matrices; GLRLM, gray level run length matrices; FOS, first-order-statistic; STD, standard deviation; MAD, mean absolute deviation; RMS, root mean square; IMOC, information measure of correlation; R, ray; D, diameter.

Feature	*P*-value	Mean IJ	Mean HJ
Acetabulum_SS_Compactness 1	**1.95 × 10^−4^**	70.532	71.172
Acetabulum_SS_Compactness 2	**2.04 × 10^−4^**	0.078	0.076
Acetabulum_SS_Maximum 3D Diameter	**7.05 × 10^−27^**	135.041	138.882
Acetabulum_SS_Minimum 3D Diameter	1.00 × 10^0^	0.000	0.000
Acetabulum_SS_Median 3D Diameter	**3.89 × 10^−8^**	80.286	81.058
Acetabulum_SS_Mean 3D Diameter	**8.40 × 10^−25^**	76.865	77.662
Acetabulum_SS_STD 3D Diameter	**3.84 × 10^−6^**	27.655	28.414
Acetabulum_SS_Variance 3D Diameter	**3.84 × 10^−6^**	781.661	820.372
Acetabulum_SS_Skewness 3D Diameter	**5.02 × 10^−4^**	−0.375	−0.363
Acetabulum_SS_Kurtosis 3D Diameter	3.08** **× 10^−1^	2.303	2.310
Acetabulum_SS_Equivalent R	**8.23 × 10^−9^**	29.685	29.940
Acetabulum_SS_Max (D/2)/R	**1.33 × 10^−6^**	2.278	2.323
Acetabulum_SS_Spherical disproportion	**2.04 × 10^−4^**	2.357	2.377
Acetabulum_SS_Sphericity	**2.04 × 10^−4^**	0.425	0.422
Acetabulum_SS_Area:	**4.12 × 10^−8^**	26,104.756	26,797.037
Acetabulum_SS_Surface to volume ratio	8.01 × 10^−1^	0.239	0.239
Acetabulum_SS_Volume	**8.23 × 10^−9^**	110,294.697	113,301.147
Acetabulum_SS_mean normal0	**5.02 × 10^−38^**	0.002	0.000
Acetabulum_SS_mean normal1	7.05 × 10^−2^	0.000	0.000
Acetabulum_SS_mean normal2	**8.90 × 10^−63^**	0.000	0.000
Acetabulum_FOS_Signal Energy	1.25 × 10^−1^	8,908,360,167.973	9,029,094,220.532
Acetabulum_FOS_Signal Kurtosis	**3.28 × 10^−8^**	3.164	3.002
Acetabulum_FOS_Signal MAD	**2.30 × 10^−4^**	45.377	45.680
Acetabulum_FOS_Signal Max	**5.92 × 10^−11^**	506.108	483.538
Acetabulum_FOS_Signal Mean	5.13** × **10**^−^**^1^	148.772	149.660
Acetabulum_FOS_Signal Median	6.60** **× 10^−1^	151.536	152.836
Acetabulum_FOS_Signal Min	**1.02 × 10^−45^**	0.103	0.027
Acetabulum_FOS_Signal Range	**6.59 × 10^−11^**	506.006	483.511
Acetabulum_FOS_Signal RMS	5.01 × 10^−1^	159.258	160.171
Acetabulum_FOS_Signal Skewness	**8.14 × 10^−4^**	−0.013	−0.067
Acetabulum_FOS_Signal STD	3.20 × 10^−2^	56.610	56.744
Acetabulum_FOS_Signal Variance	3.20 × 10^−2^	3,269.960	3,290.429
Acetabulum_FOS_Histogram Entropy	**7.44 × 10^−4^**	3.870	3.932
Acetabulum_FOS_Histogram Kurtosis	**9.43 × 10^−4^**	5.834	5.370
Acetabulum_FOS_Histogram MAD	**3.11 × 10^−7^**	0.023	0.023
Acetabulum_FOS_Histogram Max	**3.01 × 10^−6^**	0.116	0.109
Acetabulum_FOS_Histogram Mean	1.00 × 10^0^	0.016	0.016
Acetabulum_FOS_Histogram Median	**2.85 × 10^−8^**	0.000	0.000
Acetabulum_FOS_Histogram Min	1.00 × 10^0^	0.000	0.000
Acetabulum_FOS_Histogram Range	**3.01 × 10^−6^**	0.116	0.109
Acetabulum_FOS_Histogram RMS	1.57** × **10**^−^**^2^	0.035	0.034
Acetabulum_FOS_Histogram Skewness	**5.38 × 10^−3^**	2.002	1.914
Acetabulum_FOS_Histogram STD	1.57 × 10^−2^	0.032	0.031
Acetabulum_FOS_Histogram Uniformity	1.57 × 10^−2^	0.080	0.076
Acetabulum_FOS_Histogram Variance	1.57 × 10^−2^	0.001	0.001
Acetabulum_FOS_Histogram TotalFrequency	2.01 × 10^−2^	350,260.772	354,414.303
Acetabulum_FOS_Histogram Quantile 0.01	**4.60 × 10^−7^**	20.879	20.089
Acetabulum_FOS_Histogram Quantile 0.1	9.38 × 10**^−^**^1^	71.165	71.871
Acetabulum_FOS_Histogram Quantile 0.2	5.37 × 10**^−^**^1^	99.367	99.955
Acetabulum_FOS_Histogram Quantile 0.3	1.96 × 10**^−^**^1^	119.924	120.471
Acetabulum_FOS_Histogram Quantile 0.4	2.59 × 10**^−^**^1^	136.658	137.477
Acetabulum_FOS_Histogram Quantile 0.5	6.31 × 10**^−^**^1^	151.467	152.765
Acetabulum_FOS_Histogram Quantile 0.6	2.11 × 10**^−^**^1^	165.649	167.260
Acetabulum_FOS_Histogram Quantile 0.7	1.97 × 10**^−^**^1^	180.254	181.852
Acetabulum_FOS_Histogram Quantile 0.8	6.42 × 10**^−^**^1^	196.832	198.016
Acetabulum_FOS_Histogram Quantile 0.9	6.01 × 10**^−^**^1^	219.129	219.801
Acetabulum_FOS_Histogram Quantile 0.99	2.50 × 10**^−^**^1^	275.952	275.856
Acetabulum_GLCM1_Autocorrelation	**2.09 × 10^−3^**	412.618	412.658
Acetabulum_GLCM1_Cluster Prominence	**1.16 × 10^−3^**	3,785,761.635	3,762,839.933
Acetabulum_GLCM1_Cluster Shade	**1.88 × 10^−3^**	77,673.614	77,506.818
Acetabulum_GLCM1_Cluster Tendency	**2.21 × 10^−3^**	1,669.689	1,670.428
Acetabulum_GLCM1_Contrast	8.85 × 10^−1^	24.033	24.616
Acetabulum_GLCM1_Correlation	**5.59 × 10^−8^**	1,351,021.973	1,446,471.264
Acetabulum_GLCM1_Difference Entropy	5.86 × 10^−1^	3.285	3.303
Acetabulum_GLCM1_Dissimilarity	3.17 × 10^−1^	3.421	3.481
Acetabulum_GLCM1_Energy	**5.27 × 10^−17^**	0.004	0.003
Acetabulum_GLCM1_Entropy	**4.92 × 10^−9^**	8.688	8.728
Acetabulum_GLCM1_Homogeneity	6.93 × 10^−2^	0.381	0.376
Acetabulum_GLCM1_homogeneity2	4.47 × 10^−2^	0.301	0.296
Acetabulum_GLCM1_IMOC1	7.58 × 10^−2^	−0.163	−0.160
Acetabulum_GLCM1_IMOC2	2.53 × 10^−2^	0.854	0.849
Acetabulum_GLCM1_Inverse Difference moment	8.34 × 10^−1^	1.000	1.000
Acetabulum_GLCM1_Inverse Difference moment2	3.14 × 10^−1^	0.997	0.997
Acetabulum_GLCM1_Inverse Variance	7.70 × 10^−2^	0.294	0.289
Acetabulum_GLCM1_Max Probability	**5.20 × 10^−31^**	0.010	0.009
Acetabulum_GLCM1_Sum Average	3.74 × 10^−2^	38.546	38.583
Acetabulum_GLCM1_Sum Entropy	**5.62 × 10^−10^**	5.651	5.662
Acetabulum_GLCM1_Inertia	8.85 × 10^−1^	24.033	24.616
Acetabulum_GLCM1_Variance	3.74 × 10^−2^	636.010	636.624
Acetabulum_GLCM1_SumVariance	**2.32 × 10^−3^**	1,270.949	1,270.749
Acetabulum_GLRLM1_Short Run Emphasis	1.00 × 10^0^	1.000	1.000
Acetabulum_GLRLM1_Long Run Emphasis	1.00 × 10^0^	1.000	1.000
Acetabulum_GLRLM1_Gray Level Non Uniformity	1.33 × 10^−1^	10,131.157	10,161.108
Acetabulum_GLRLM1_Run Length Non Uniformity	1.88 × 10^−2^	243,991.016	248,399.650
Acetabulum_GLRLM1_Run Percentage	2.21 × 10^−2^	0.700	0.701
Acetabulum_GLRLM1_Low Gray Level Run Emphasis	**3.48 × 10^−08^**	0.008	0.008
Acetabulum_GLRLM1_high Gray Level Run Emphasis	4.57 × 10^−1^	488.358	491.584
Acetabulum_GLRLM1_Short Run Low Gray Level Emphasis	**3.48 × 10^−8^**	0.008	0.008
Acetabulum_GLRLM1_Short Run High Gray Level Emphasis	4.57 × 10^−1^	488.358	491.584
Acetabulum_GLRLM1_Long Run Low Gray Level Emphasis	**3.48 × 10^−8^**	0.008	0.008
Acetabulum_GLRLM1_Long Run High Gray Level Emphasis	4.57 × 10^−1^	488.358	491.584
Femur_SS_Compactness 1	**2.80 × 10^−5^**	97.238	95.325
Femur_SS_Compactness 2	**4.06 × 10^−5^**	0.311	0.320
Femur_SS_Maximum 3D Diameter	**1.28 × 10^−11^**	98.059	96.502
Femur_SS_Minimum 3D Diameter	1.00 × 10^0^	0.000	0.000
Femur_SS_Median 3D Diameter	**1.32 × 10^−32^**	52.943	51.766
Femur_SS_Mean 3D Diameter	**1.58 × 10^−28^**	53.273	52.292
Femur_SS_STD 3D Diameter	**3.99 × 10^−3^**	21.963	21.716
Femur_SS_Variance 3D Diameter	**3.99 × 10^−3^**	485.407	474.968
Femur_SS_Skewness 3D Diameter	**5.95 × 10^−6^**	−0.003	0.014
Femur_SS_Kurtosis 3D Diameter	**2.91 × 10^−5^**	2.029	2.017
Femur_SS_Equivalent R	**1.55 × 10^−15^**	29.942	29.468
Femur_SS_Max (D/2)/R	5.95 × 10^−2^	1.640	1.638
Femur_SS_Spherical disproportion	**4.06 × 10^−5^**	1.488	1.470
Femur_SS_Sphericity	**4.06 × 10^−5^**	0.675	0.682
Femur_SS_Area:	**5.41 × 10^−18^**	16,812.378	16,063.082
Femur_SS_Surface to volume ratio	8.92 × 10^−1^	0.150	0.150
Femur_SS_Volume	**1.56 × 10^−15^**	113,467.792	107,716.557
Femur_SS_mean normal0	3.32 × 10^−2^	0.000	0.001
Femur_SS_mean normal1	1.70 × 10^−2^	0.000	0.000
Femur_SS_mean normal2	**1.43 × 10^−7^**	0.007	0.004
Femur_FOS_Signal Energy	6.00 × 10^−1^	5,452,299,893.617	5,413,605,031.369
Femur_FOS_Signal Kurtosis	**1.57 × 10^−5^**	3.189	3.150
Femur_FOS_Signal MAD	**9.01 × 10^−9^**	44.042	45.469
Femur_FOS_Signal Max	**1.52 × 10^−3^**	474.457	471.013
Femur_FOS_Signal Mean	5.14 × 10^−2^	107.595	109.316
Femur_FOS_Signal Median	9.57 × 10^−1^	103.399	105.017
Femur_FOS_Signal Min	**6.17 × 10^−4^**	0.005	0.005
Femur_FOS_Signal Range	**1.51 × 10^−3^**	474.452	471.008
Femur_FOS_Signal RMS	**9.18 × 10^−3^**	120.138	122.498
Femur_FOS_Signal Skewness	6.08 × 10^−2^	0.492	0.460
Femur_FOS_Signal STD	**1.04 × 10^−11^**	53.108	54.840
Femur_FOS_Signal Variance	**1.04 × 10^−11^**	2,901.252	3,096.223
Femur_FOS_Histogram Entropy	**1.22 × 10^−8^**	3.757	3.818
Femur_FOS_Histogram Kurtosis	**1.94 × 10^−7^**	7.490	6.996
Femur_FOS_Histogram MAD	**7.29 × 10^−5^**	0.023	0.023
Femur_FOS_Histogram Max	**6.14 × 10^−8^**	0.141	0.135
Femur_FOS_Histogram Mean	1.00 × 10^0^	0.016	0.016
Femur_FOS_Histogram Median	6.13 × 10^−1^	0.000	0.000
Femur_FOS_Histogram Min	1.00 × 10^0^	0.000	0.000
Femur_FOS_Histogram Range	**6.14 × 10^−8^**	0.141	0.135
Femur_FOS_Histogram RMS	**3.78 × 10^−11^**	0.037	0.036
Femur_FOS_Histogram Skewness	**4.04 × 10^−8^**	2.243	2.130
Femur_FOS_Histogram STD	**3.78 × 10^−11^**	0.034	0.033
Femur_FOS_Histogram Uniformity	**3.78 × 10^−11^**	0.088	0.084
Femur_FOS_Histogram Variance	**3.77 × 10^−11^**	0.001	0.001
Femur_FOS_Histogram TotalFrequency	**5.28 × 10^−6^**	364,675.636	341,770.827
Femur_FOS_Histogram Quantile 0.01	**2.74 × 10^−32^**	11.652	10.566
Femur_FOS_Histogram Quantile 0.1	**4.94 × 10^−11^**	42.755	41.529
Femur_FOS_Histogram Quantile 0.2	8.77 × 10^−2^	57.505	58.061
Femur_FOS_Histogram Quantile 0.3	6.83 × 10^−1^	72.388	73.532
Femur_FOS_Histogram Quantile 0.4	2.71 × 10^−1^	87.956	89.458
Femur_FOS_Histogram Quantile 0.5	9.79 × 10^−1^	103.491	105.169
Femur_FOS_Histogram Quantile 0.6	9.29 × 10^−1^	119.544	121.615
Femur_FOS_Histogram Quantile 0.7	5.92 × 10^−2^	136.633	139.423
Femur_FOS_Histogram Quantile 0.8	**3.96 × 10^−3^**	155.352	158.833
Femur_FOS_Histogram Quantile 0.9	**4.43 × 10^−4^**	178.631	182.855
Femur_FOS_Histogram Quantile 0.99	3.54 × 10^−2^	236.499	240.235
Femur_GLCM1_Autocorrelation	1.33 × 10^−1^	262.482	266.457
Femur_GLCM1_Cluster Prominence	1.40 × 10^−1^	2,025,184.196	2,086,235.334
Femur_GLCM1_Cluster Shade	7.53 × 10^−2^	44,524.817	45,592.806
Femur_GLCM1_Cluster Tendency	9.93 × 10^−2^	1,064.219	1,080.678
Femur_GLCM1_Contrast	**2.55 × 10^−4^**	17.938	18.510
Femur_GLCM1_Correlation	**1.40 × 10^−3^**	1,088,137.899	1,292,560.837
Femur_GLCM1_Difference Entropy	**2.07 × 10^−3^**	3.033	3.057
Femur_GLCM1_Dissimilarity	**1.72 × 10^−3^**	2.800	2.852
Femur_GLCM1_Energy	**2.88 × 10^−5^**	0.005	0.005
Femur_GLCM1_Entropy	**6.48 × 10^−6^**	8.410	8.456
Femur_GLCM1_Homogeneity	**3.75 × 10^−3^**	0.431	0.427
Femur_GLCM1_homogeneity2	**1.77 × 10^−3^**	0.360	0.355
Femur_GLCM1_IMOC1	9.06 × 10^−1^	−0.216	−0.216
Femur_GLCM1_IMOC2	8.47 × 10^−1^	0.911	0.910
Femur_GLCM1_Inverse Difference moment	**3.88 × 10^−4^**	1.000	1.000
Femur_GLCM1_Inverse Difference moment2	**1.68 × 10^−3^**	0.997	0.997
Femur_GLCM1_Inverse Variance	**6.76 × 10^−8^**	0.339	0.332
Femur_GLCM1_Max Probability	2.26 × 10^−2^	0.016	0.017
Femur_GLCM1_Sum Average	1.58 × 10^−1^	29.192	29.319
Femur_GLCM1_Sum Entropy	**1.31 × 10^−4^**	5.648	5.672
Femur_GLCM1_Inertia	**2.55 × 10^−4^**	17.938	18.510
Femur_GLCM1_Variance	1.58 × 10^−1^	481.675	483.756
Femur_GLCM1_SumVariance	9.09 × 10^−2^	768.788	782.895
Femur_GLRLM1_Short Run Emphasis	1.00 × 10^0^	1.000	1.000
Femur_GLRLM1_Long Run Emphasis	1.00 × 10^0^	1.000	1.000
Femur_GLRLM1_Gray Level Non Uniformity	**1.79 × 10^−7^**	11,202.527	10,159.714
Femur_GLRLM1_Run Length Non Uniformity	**1.27 × 10^−5^**	274,901.209	254,372.230
Femur_GLRLM1_Run Percentage	6.58 × 10^−1^	0.768	0.762
Femur_GLRLM1_Low Gray Level Run Emphasis	**6.18 × 10^−24^**	0.015	0.017
Femur_GLRLM1_high Gray Level Run Emphasis	1.56 × 10^−1^	329.245	333.812
Femur_GLRLM1_Short Run Low Gray Level Emphasis	**6.18 × 10^−24^**	0.015	0.017
Femur_GLRLM1_Short Run High Gray Level Emphasis	1.56 × 10^−1^	329.245	333.812
Femur_GLRLM1_Long Run Low Gray Level Emphasis	**6.18 × 10^−24^**	0.015	0.017
Femur_GLRLM1_Long Run High Gray Level Emphasis	1.56 × 10^−1^	329.245	333.812

For each femur and acetabulum ROI, the 12 signal FOS features were extracted from the water-only 3D-FLASH grayscale image values in the ROIs. The following 25 histogram FOS features described the complexity of the shape of the histogram distribution of the grayscale values in the ROIs. The histogram settings for all feature classes were set to 32 bins with a marginal scale of 0.5 and minimum and maximum equal to 0 and 200, respectively. These first two subsets of features belonged to the FOS features. Texture features were based on the gray level co-occurrence matrices (GLCM) and gray level run length matrices (GLRLM) (27), calculated in 26 directions, one for every neighbor of a voxel in a 3D space with a radius set to one pixel. For each GLCM and GLRLM feature, the extracted features were averaged over the 26 directions to get 23 GLCM features and 11 GLRLM features per ROI. Lastly, 20 shape and size features were extracted from the ROI mesh of the femur and acetabulum separately.

The resulting 182 features were subdivided in 24 subsets with a variable number of features, divided by feature type and femur or acetabulum ROI. For each subset, a univariate ANOVA *F*-value analysis was applied to find the five most pertinent features based on *p*-values among those included. This yielded 24 additional *F*-contrast subsets with five features each, for a total of 48 subsets. The feature selection was repeated 100 times using 90% of the dataset and used the five most frequent features selected by the *F*-contrast rank.

### 2.4. Machine learning model training and evaluation

A K-nearest neighbor machine learning model was used to identify the features most pertinent to differentiate IJs from HJs. From the available data, 240 augmented datasets consisting of two HJs and two IJs were randomly selected as a hold-out testing dataset for model evaluation. The remaining 1,980 augmented datasets consisting of 900 datasets from 15 IJs and 1,080 datasets from 18 HJs were used for model training and validation. For each of the 48 feature subsets, a K-nearest neighbor model (*k* = 3) was trained and validated using 100-fold cross-validation with a 75/25 data split. During this selection process, the augmented images of one patient belonged only to one group either training or testing. The inputs of each model were the *z*-scored values of the radiomic features in the corresponding subset, and the outputs were the labels HJ and IJ. The trained model with the highest prediction accuracy was selected as the final model for the particular subset of features and was evaluated against the hold-out testing dataset to assess its performance in differentiating IJs from HJs. The process resulted in one trained model for each of the 48 subsets of features which was then evaluated against the testing dataset.

## 3. Results

Table 1 shows the mean values of each feature distribution in the femur and acetabulum for HJs and IJs and the corresponding *p*-values for the Wilcoxon rank sum tests comparing differences in values between groups. The results show that 116 features out of the total 182 features could differentiate IJs from HJs (*p* < 0.05, hereinafter indicated by *). Out of these 116 features, 45 features (39%) belonged to the intensity-based FOS group [16 signal (14%) and 29 histogram (25%)], 33 (28%) to the shape and size group, and 38 (33%) to the textural features group [28 GLCM (24%) and 10 GLRLM (9%)]. Among the 45 statistically significant FOS features, 24 features were from the femur (8 signal and 16 histograms), 21 from the acetabulum (8 signal and 13 histogram), and 20 were from both the femur and acetabulum (8 signal and 12 histogram).

Table 2 shows the diagnostic performance of the machine learning models for differentiating IJs from HJs using the hold-out testing dataset. For each subset of features, the accuracy, specificity, sensitivity, and AUC of the models were reported along with the number of features in the training subset. The table had 48 entries, 24 reporting the performance of the model trained using all the features in a specific subset and 24 entries reporting the performance of the model trained using only the five most pertinent features in the specific subset with the lowest *F*-contrast *p*-values. The top performing models analyzed all GLCM texture features from the femur and acetabulum followed by the models analyzing all intensity-based FOS features from the femur and acetabulum, all shape and size features from the femur and acetabulum, and all intensity-based histogram FOS features of the femur.

**Table 2:** Diagnostic performance of the machine learning models for differentiating IJs from HJs using each subset of features on the hold-out testing dataset. The complete list of features contained in each subsets can be seen in Supplementary Table S1.

Subset	Features Selection	Accuracy	Specificity	Sensitivity	AUC	# Features
Acetabulum	All	0.963	0.965	0.962	0.963	91
Acetabulum	*F*-contrast	0.966	0.961	0.971	0.966	5
Acetabulum_FOS	All	0.962	0.960	0.964	0.962	37
Acetabulum_FOS	*F*-contrast	0.900	0.901	0.898	0.900	5
Acetabulum_GLCM	All	0.962	0.969	0.955	0.962	23
Acetabulum_GLCM	*F*-contrast	0.967	0.965	0.969	0.967	5
Acetabulum_GLRLM	All	0.961	0.965	0.956	0.961	11
Acetabulum_GLRLM	*F*-contrast	0.953	0.956	0.948	0.952	5
Acetabulum_Histogram	All	0.953	0.961	0.943	0.952	25
Acetabulum_Histogram	*F*-contrast	0.910	0.923	0.894	0.908	5
Acetabulum_Signal	All	0.964	0.964	0.964	0.964	12
Acetabulum_Signal	*F*-contrast	0.952	0.970	0.931	0.950	5
Acetabulum_SS	All	0.960	0.956	0.964	0.960	20
Acetabulum_SS	*F*-contrast	0.956	0.959	0.952	0.955	5
Acetabulum_Texture	All	0.961	0.962	0.960	0.961	34
Acetabulum_Texture	*F*-contrast	0.959	0.961	0.957	0.959	5
ALL	All	0.976	0.980	0.971	0.975	182
ALL	*F*-contrast	0.954	0.959	0.948	0.954	5
Femur	All	0.977	0.977	0.976	0.977	91
Femur	*F*-contrast	0.935	0.940	0.929	0.935	5
Femur_FOS	All	0.969	0.971	0.965	0.968	37
Femur_FOS	*F*-contrast	0.960	0.954	0.966	0.960	5
Femur_GLCM	All	0.971	0.977	0.964	0.971	23
Femur_GLCM	*F*-contrast	0.971	0.973	0.969	0.971	5
Femur_GLRLM	All	0.973	0.979	0.965	0.972	11
Femur_GLRLM	*F*-contrast	0.922	0.924	0.920	0.922	5
Femur_Histogram	All	0.970	0.973	0.965	0.969	25
Femur_Histogram	*F*-contrast	0.953	0.951	0.956	0.953	5
Femur_Signal	All	0.972	0.969	0.975	0.972	12
Femur_Signal	*F*-contrast	0.969	0.969	0.969	0.969	5
Femur_SS	All	0.915	0.921	0.908	0.915	20
Femur_SS	*F*-contrast	0.903	0.910	0.896	0.903	5
Femur_Texture	All	0.968	0.975	0.960	0.967	34
Femur_Texture	*F*-contrast	0.965	0.973	0.955	0.964	5
FOS	All	0.972	0.975	0.969	0.972	74
FOS	*F*-contrast	0.949	0.955	0.941	0.948	5
GLCM	All	0.977	0.977	0.976	0.977	46
GLCM	*F*-contrast	0.972	0.977	0.966	0.972	5
GLRLM	All	0.976	0.980	0.972	0.976	22
GLRLM	*F*-contrast	0.903	0.922	0.881	0.902	5
Histogram	All	0.966	0.967	0.964	0.965	50
Histogram	*F*-contrast	0.952	0.949	0.956	0.952	5
Signal	All	0.975	0.970	0.982	0.976	24
Signal	*F*-contrast	0.948	0.957	0.936	0.947	5
SS	All	0.970	0.968	0.972	0.970	40
SS	*F*-contrast	0.957	0.958	0.955	0.957	5
Texture	All	0.976	0.980	0.971	0.975	68

The model trained with all GLCM texture features from the femur and acetabulum had the highest diagnostic performance for differentiating IJs from HJs with 0.977 accuracy, 0.977 specificity, 0.976 sensitivity, and 0.977 AUC. Three of the five features of this model with the lowest *F*-contrast *p*-values were related to GLCM of the femur (GLCM Max Probability*, GLCM1 Energy*, and GLCM1 Correlation*), while two were related to GLCM of the acetabulum (GLCM Correlation*, GLCM Inverse Variance). The *F*-contrast model using the five most pertinent features had 0.972 accuracy, 0.977 specificity, 0.966 sensitivity, and 0.972 AUC.

The model trained with all FOS features from the femur and acetabulum had 0.972 accuracy, 0.975 specificity, 0.969 sensitivity, and 0.972 AUC for differentiating IJs from HJs. The five most pertinent features of this model with the lowest *F*-contrast *p*-value were all related to the histogram of the femur (Histogram Quantile 0.99, Histogram Quantile 0.6, Histogram Uniformity*, Histogram Quantile 0.4, Histogram RMS*). The *F*-contrast model using these five features had 0.949 accuracy, 0.955 specificity, 0.941 sensitivity, and 0.948 AUC.

The model trained with all shape and size features from the femur and acetabulum had 0.970 accuracy, 0.968 specificity, 0.972 sensitivity, and 0.970 AUC for differentiating IJs from HJs. The five most pertinent features of this model with the lowest *F*-contrast *p*-values were all related to the shape and size of the femur (SS Area*, SS Mean 3D Diameter*, SS Median 3D Diameter*, Equivalent R*). The *F*-contrast model using these five features had 0.957 accuracy, 0.958 specificity, 0.955 sensitivity, and 0.957 AUC. As shown in Table 1, among the 40 shape and size features of the femur and acetabulum, 33 (83%) were significantly different between HJs and IJs.

The models trained with all intensity-based FOS histogram features from the femur had 0.972 accuracy, 0.969 specificity, 0.975 sensitivity, and 0.972 AUC for differentiating IJs from HJs. The five most pertinent features of this model with the lowest *F*-contrast *p*-values included the Femur Histogram Quantile 0.1*, Femur Histogram Total Frequency*, Femur Histogram Median, Femur Histogram Range*, and Femur Histogram Quantile 0.3.) The *F*-contrast model using these five features had 0.953 accuracy, 0.951 specificity, 0.956 sensitivity, and 0.953 AUC.

The model trained with the femur histogram features yielded an accuracy of 0.97 (0.97, 0.973, 0.965, 0.969). For the subset with the five most relevant features (*F*-contrast), these values became 0.953, 0.951, 0.956, and 0.953. In particular in the *F*-contrast subset included the femur Histogram Quantile 0.1*, femur Histogram Total frequency*, femur Histogram Median, femur Histogram Range*, and femur Histogram Quantile 0.3 features.

**Figure 3:**
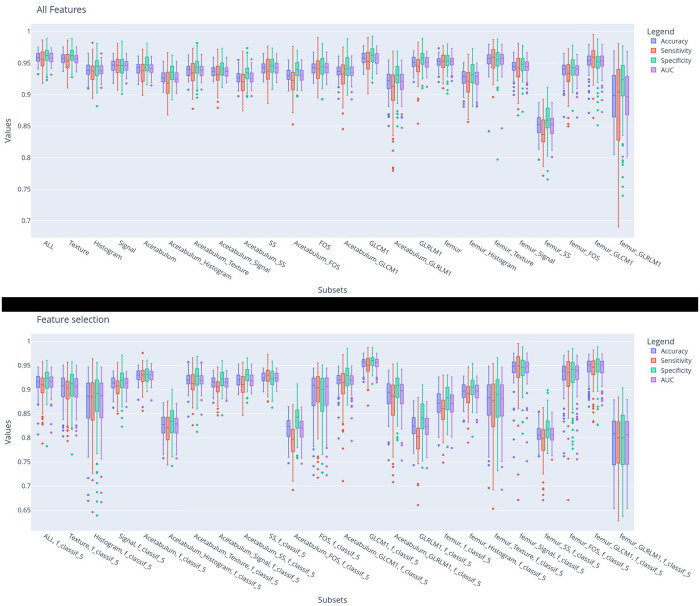
Diagnostic performance of the machine learning models for differentiating IJs from HJs using each subset of features during model training. The histogram bars represent the distribution of the prediction metrics during the 100-fold cross-validation.

Figure 3 shows the diagnostic performance of the machine learning models for differentiating IJs from HJs during the 100-fold cross-validation training phase. Models trained with femur intensity-based FOS and GLCM texture features all had accuracies above 0.95, while most models trained with acetabular intensity-based FOS and GLCM texture features had accuracies under 0.95. The differences were more notable for the *F*-contrast models trained using the five most pertinent features with the lowest *F*-contrast *p*-values, where three of the four models with the highest accuracy used features from the femur. As shown in Table 2, differences in model performance were also confirmed using the hold-out testing dataset, where the model trained with 91 features from the femur had higher diagnostic performance (0.977 accuracy, 0.977 sensitivity, 0.976 specificity, and 0.977 AUC) when compared to models trained with all 182 features from the femur and acetabulum (0.976 accuracy, 0.980 specificity, 0.971 sensitivity, and 0.975 AUC) and models trained with 91 features from the acetabulum (0.963 accuracy, 0.965 specificity, 0.962 sensitivity, and 0.963 AUC). In particular, the model trained with the femur had higher accuracy compared to the ones trained with the acetabulum ones (Rank-sum test *p* < 0.05) even in the *F*-contrasted subset (bottom subplot).

**Figure 4:**
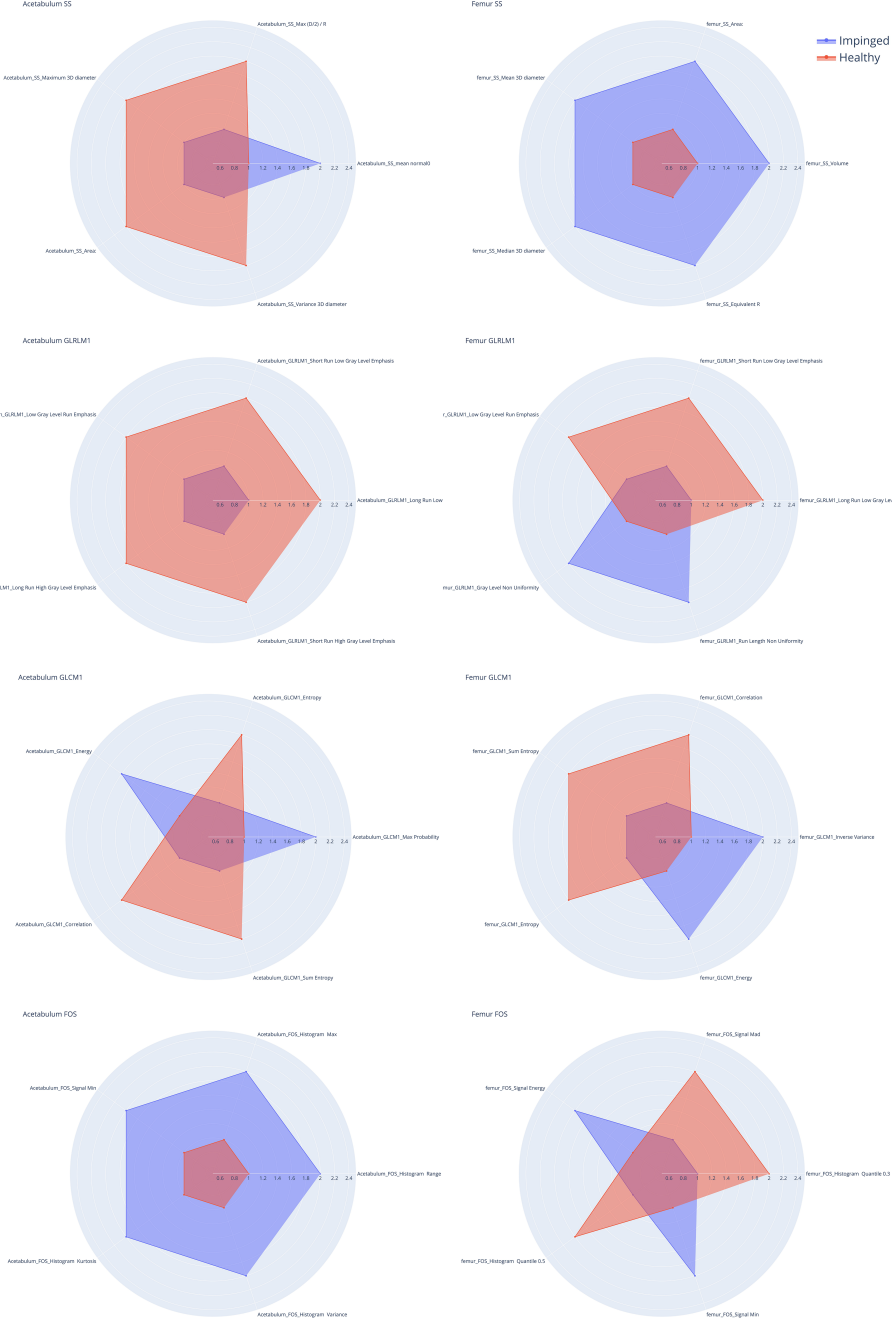
Radar charts for shape and size (SS), gray level Co-occurrence matrix (GLCM), gray level Run matrix (GLRLM) and intensity based first order statistic (FOS) of the acetabulum (left) and the femur (right). The five spokes represent the five most informative features in the group (*F*-contrast), the radial length of each spoke is proportional to the magnitude of the value of the associated feature. The spokes are normalized so that the difference between hip joints with impingement (IJ, blue line) and the healthy ones (HJ, orange line) is emphasized. For example, in the SS Acetabulum radar plot it is possible to see how four features values are higher for the healthy joints compared to the injured ones (first plot on the left) while the mean normal0 features values are higher in the injured acetabulum than in the healthy ones.

**Figure 5:**
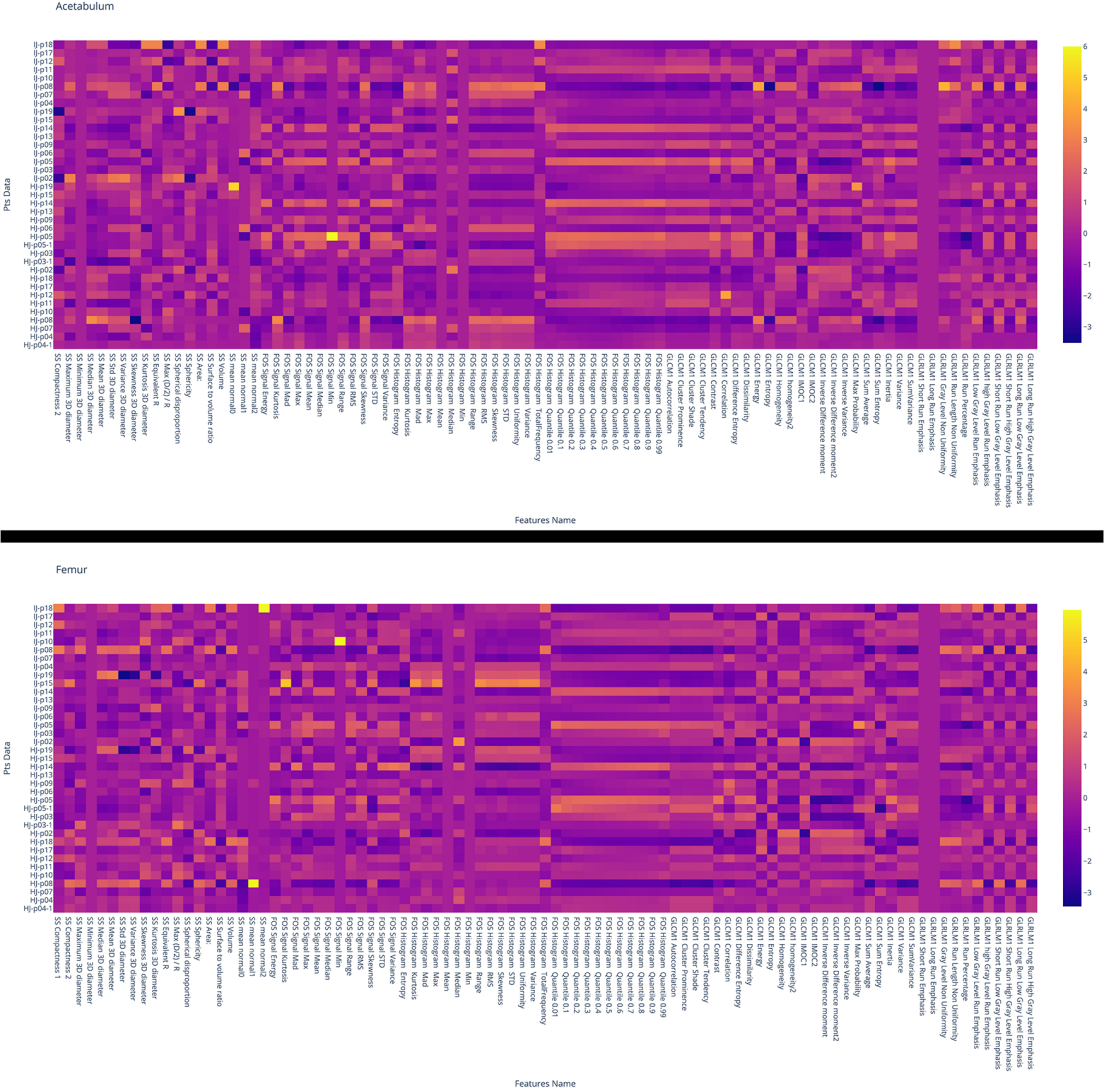
Heat map of the values of the features for the acetabulum (top) and femur (bottom). Each row corresponds to one patient and each column corresponds to one normalized (*z*-score) radiomic feature. HJ or IJ before the patient number refers to a healthy joint and joint with impingement, respectively. From the heat map it is possible to see how both femur and acetabulum GLCM Correlation feature is of higher value for HJ than IJ.

Figure 4 shows the five most pertinent features with the lowest *F*-contrast *p*-values for each feature class, while Figure 5 shows *z*-scored values of each feature for the femur and acetabulum. For the femur, the five most pertinent features were three textural features (GLRLM Long Run Low Gray Level Emphasis*, GLRLM Short Run Low Gray Level Emphasis*, and GLRLM Low Gray Level Run Emphasis*) and two shape and size features (SS Area*, SS Volume*). The values of the three GLRLM features of the femur and the area and volume of the femur were higher in IJs than HJs. The importance of the three GLRLM features of the femur were further confirmed by the results in Table 2; Supplementary Table S1, which showed that the five most pertinent features with the lowest *F*-contrast *p*-values in the model trained with all 182 features from the femur and acetabulum included GLRLM Long Run Low Gray Level Emphasis*, GLRLM Short Run Low Gray Level Emphasis*, and GLRLM Low Gray Level Run Emphasis* of the femur.

## 4. Discussion and conclusions

Our study was performed to investigate the feasibility of using radiomics of 3D-MRI to distinguish between hips with and without symptomatic impingement in patients with FAI. Our results showed some of the highest diagnostic performance for differentiating IJs from HJs using imaging studies reported in the literature. The top performing radiomic model in our study analyzed all GLCM texture features from the femur and acetabulum on 3D-MRI, followed by models analyzing all intensity-based FOS features from the femur and acetabulum, all shape and size features from the femur and acetabulum, and all histogram FOS features of the femur.

FAI is characterized by impingement of the femoral head-neck junction against the acetabular rim due to morphologic abnormalities of the proximal femur and acetabulum (2–4). In our study, the radiomic model trained with all shape and size features from the femur and acetabulum on 3D-MRI yielded the highest performance with 0.970 accuracy, 0.968 specificity, 0.972 sensitivity, and 0.970 AUC for differentiating IJs from HJs. The model had higher diagnostic performance for detecting FAI than currently used quantitative measures of bone shape on radiographs, CT, and MRI. For example, studies have shown that the alpha angle has sensitivities between 0.360 and 0.920 and specificities between 0.620 and 0.950 for detecting cam impingement (28–32), while the center edge angle has sensitivities between 0.820 and 0.842 and specificities between 0.390 and 1.00 for detecting pincer impingement (32, 33). Furthermore, a high prevalence of abnormal quantitative measures of proximal femur and acetabulum shape have been described in healthy subjects with no clinical evidence of FAI, which raises questions regarding the high specificities of these metrics reported in some studies (34).

Although FAI is a condition caused by morphological abnormalities of bone, our study found that the radiomic model analyzing all GLCM texture features of the femur and acetabulum on 3D-MRI had the highest diagnostic performance for differentiating IJs from HJs. GLCM features are calculated over the co-occurrence matrix, which highlights how spread out the image pixel signal intensity values are around a given pixel in a square matrix. If all the pixels in the ROI had the same grayscale value (i.e., pixel signal intensity values were homogeneous), the co-occurrence matrix would have only one bin containing that particular co-occurrence image intensity value set to 1 and all the other bins set to 0. The presence of multiple peaks in the co-occurrence implies heterogeneity in image pixel signal intensity. If the imaged tissue is mildly heterogeneous, the values in the co-occurrence matrix are less parse and more close to each other, whereas if the pixel values are completely random, the co-occurrence matrix will have sparser peaks (27). In our study, the model trained with all GLCM texture features from the femur and acetabulum had 0.977 accuracy, 0.977 specificity, 0.976 sensitivity, and 0.977 AUC for distinguishing between IJs and HJs. The five most pertinent features of this model were GLCM Max Probability, GLCM1 Energy, and GLCM1 Correlation of the femur and GLCM Correlation and GLCM Inverse Variance of the acetabulum. All these features were higher in the IJ than the HJ, indicating that FAI leads to a more heterogeneous distribution of image pixel signal intensity values. The femur and acetabulum primarily consist of trabecular and cortical bone, hematopoietic cells, and fat with little if any water content. As the water-only 3D-FLASH images used for radiomic analysis in our study reflect the presence of water within each image pixel, the greater heterogeneity of pixel signal intensity values in the IJs likely results in increased water content in some pixels. This may be due to subtle and non-uniform bone inflammation due to impingement of the femoral head-neck junction against the acetabular rim, which cannot even be detected in the image by the human eye.

Our study has shown that it is possible to create machine learning models to differentiate IJ from HJ with a high diagnostic performance using only a small subset of radiomic features on 3D-MRI. For each feature class, there was a relatively small decrease in model performance when using the five most pertinent features with the lowest *F*-contrast *p*-values compared to the full model analyzing all features from the femur and acetabulum. For example, the *F*-contrast model for GLCM texture features had 0.972 accuracy, 0.977 specificity, 0.966 sensitivity, and 0.972 AUC for differentiating IJs from HJs compared to 0.977 accuracy, 0.977 specificity, 0.976 sensitivity, and 0.977 for the full model. Radiomic models analyzing a smaller number of features are better suited for widespread use in clinical practice as they are quicker and easier to create and are likely more reproducible across different MRI scanners, sequences, and imaging parameters.

Our study had several limitations. One limitation was the small number of subjects used for model training and evaluation. The problem of model training with a small number of subjects was overcome by using a novel data augmentation framework to create pseudo-plausible image data that magnified the pattern in the features space between the IJs and HJs. Furthermore, our models were created using a simple K-nearest neighbor method to focus attention on the information content of the image features rather than the accuracy of the models *per se*. However, the relative simplicity of our machine learning approach may improve the reproducibility of the models and indirectly determines the lower bound of model performance as sensitivity and specificity could likely be improved with use of more sophisticated machine learning methods and larger training datasets. A final limitation was that our study could not assess model generalizability as model training and evaluation was performed using homogenous image datasets acquired on the same MRI scanner with the same sequence and imaging parameters.

In conclusion, our study has documented the feasibility of using radiomics of 3D-MRI to distinguish between hips with and without symptomatic impingement in patients with FAI. Our radiomic models analyzed intensity-based FOS features, shape and size features, and texture features and had some of the highest diagnostic performance for differentiating IJs from HJs using imaging studies reported in the literature. Additional studies are needed to investigate the use of more sophisticated machine learning approaches and larger training datasets to optimize model performance and to evaluate model generalizability using more heterogeneous patient populations imaged with different MRI scanners and imaging protocols.

## Data Availability

The raw data supporting the conclusions of this article will be made available by the authors, without undue reservation.
